# ΔNp63 maintains the fidelity of the myoepithelial cell lineage and directs cell differentiation programs in the murine salivary gland

**DOI:** 10.1038/s41418-022-01101-0

**Published:** 2022-12-16

**Authors:** Eun-Ah Christine Song, Monika Che, Jason Osinski, Kirsten Smalley, Erich Horeth, Satrajit Sinha, Rose-Anne Romano

**Affiliations:** 1 Department of Oral Biology, School of Dental Medicine, State University of New York at Buffalo, Buffalo, NY USA; 2 Department of Biochemistry, Jacobs School of Medicine and Biomedical Sciences, State University of New York at Buffalo, Buffalo, NY USA

**Keywords:** Gene expression, Gene regulation

## Abstract

Salivary glands consist of several epithelial cell types of distinct lineages and functional characteristics that are established by directed differentiation programs of resident stem and progenitor cells. We have shown that ΔNp63, a crucial transcriptional regulator of stem/progenitor cells, is enriched in both the basal and myoepithelial cell (MEC) populations and that ΔNp63 positive cells maintain all the descendent epithelial cell lineages of the adult mouse salivary glands (mSGs). Although this pivotal role of ΔNp63 in driving the broader epithelial cell fate and identity in the mSG has been demonstrated, how ΔNp63 functions specifically in the commitment and differentiation of the MEC population is less understood. Using multiple genetic mouse models that allow for cell tracing, we show that ΔNp63 is critical in maintaining and renewing MECs, in part through the transcriptional regulation of *Acta2* gene expression, a defining marker of this cell population. We demonstrate that during adult mSG homeostasis, ΔNp63 enriched MECs function as bipotent progenitor cells that maintain not only the MEC population, but also the distinctly different ductal cell lineages. The fidelity of this process is dependent on ΔNp63 expression, since MEC-specific ablation of ΔNp63 results in altered MEC differentiation and affects cellular plasticity resulting in aberrant differentiation of the intercalated ducts and acinar cells. In contrast, we find that the contribution of MECs to ductal and acinar cell regeneration following severe injury is independent of ΔNp63. Our observations offer new insights into cellular mechanisms driving MEC fate choices and differentiation programs in the context of salivary gland homeostasis and in response to injury and regeneration. Long term, these findings have implications for better treatment of salivary gland dysfunction through stem cell-based approaches.

## Introduction

Salivary glands (SGs) are exocrine organs that function to produce and secrete saliva into the oral cavity, which is critical for proper speech, mastication, swallowing, and maintaining overall oral health. In humans and rodents, saliva is produced by three major pairs of glands, the parotid gland (PG), the sublingual gland (SLG), and the submandibular gland (SMG). While each of the three glands vary in the types of saliva secretions that they produce, serous or mucous, they share common cellular and molecular characteristics. The mouse SMG, a commonly used experimental model, is comprised of several epithelial cell types including acinar cells, which are the main secretory units of the gland and function to produce saliva. Myoepithelial cells (MECs), which surround the acini, aid the contractile extrusion of the saliva into the oral cavity via a well-developed ductal network formed by intercalated, granular, striated and excretory ducts [1–3]. SGs undergo repair and regeneration and a better understanding of the underlying mechanisms driving these processes have important therapeutic implications. This is particularly relevant as many systemic diseases and disorders of the SG exist that compromise the glands and their surrounding tissue, resulting in glandular hypofunction. This is best exemplified by irradiation treatment for head and neck cancers, autoimmune disorders such as Sjögren’s Syndrome, and aging [4–6]. SG dysfunction frequently leads to chronic hyposalivation, or dry mouth, which is accompanied by a difficulty in swallowing and speaking, as well as increased oral infections and dental caries, all of which can decrease patient quality of life. Unfortunately, current treatment options for hyposalivation are limited and only provide temporary relief.

Over the last several years there has been a renewed push towards identifying mechanisms of SG homeostasis and regeneration in response to injury. In this regard, genetic lineage tracing has begun to shed light on cell fate specification patterns during development, gland maintenance, repair, and regeneration. Indeed, these studies have demonstrated important roles for Keratin 14 (K14), K5, Axin2, p63, alpha-smooth muscle actin (SMA) and Kit positive cells in maintaining various ductal and myoepithelial cell populations during homeostasis [7–13]. Additionally, similar lineage tracing studies have shown that Mist1, Pip, and Sox2 positive cells in the acini maintain this important cellular population [14–16]. Interestingly, there is growing evidence that the lineage-restricted states of these cell types under normal conditions are often bypassed upon injury, where cells display increased cellular plasticity and multipotency under specific stimuli [17, 18]. This, for example, has been shown in experiments performed using the well-established ligature-induced injury model where upon mild injury, in which only the main duct is ligated, Kit, K5 and Axin2-marked cells remain lineage-restricted and contributed to ductal cell regeneration [13, 19]. Similarly, Mist1 cells remain lineage-restricted and maintain the acinar cell population following mild injury [14]. However, upon severe injury where the duct and associated vasculature is ligated, Kit, K14 and Axin2 marked ductal cells lose their lineage restriction and instead exhibit increased cellular plasticity by contributing to acinar cell regeneration [19]. Similar studies in MECs using the cell-type specific marker SMA (encoded by the *Acta2* gene) have shown these cells to assume new functional identities and are a major source contributing to acinar cell regeneration [19]. Intriguingly, MECs express high levels of the master transcriptional regulator p63, suggesting that this factor may also play an important role in MEC function.

p63, specifically the ΔNp63 isoform, is a lineage-specific transcription factor that is highly expressed in epithelial rich tissues where it plays critical roles in stem cell self-renewal, morphogenesis, and directing differentiation programs [11, 20–30]. During embryonic SMG development, ΔNp63 is expressed in the epithelial cells of the developing placode, where it plays an essential role in directing SMG cell fates as animals with targeted deletion of this isoform display a complete block in gland morphogenesis [11, 29]. This is further exemplified by lineage tracing studies which have demonstrated that during morphogenesis, ΔNp63 cells are multipotent and give rise to all SMG epithelial cell types [11]. Similarly, in adult glands, ΔNp63 expression is restricted to the basal and MECs where these ΔNp63 positive (ΔNp63^+^) cells maintain their multipotency and contribute to the basal, myoepithelial, ductal, and some acinar cells [11]. We have shown that deletion of p63 in both basal and MECs resulted in a loss of the stem/progenitor cell population and skewed differentiation which is mediated by Follistatin-dependent dysregulated TGF-β/Activin signaling. However, additional studies aimed at teasing out the more nuanced cell type-specific function of p63 in the SMG, are lacking.

To obtain a better understanding of the function of ΔNp63 in driving cell fate choices in the MECs of the SMG, we have generated animals with targeted deletion of ΔNp63 in the MECs of adult glands and have performed lineage tracing analyses. We demonstrate that during homeostasis, ablation of ΔNp63 results in the exhaustion of the MECs, highlighting the importance of this transcription factor in maintaining/renewing this cell lineage. In vivo lineage tracing experiments of the SMG MECs show that under homeostatic conditions these cells function as a bipotent stem/progenitor cell population that maintain the myoepithelial and ductal cell lineages. Moreover, we show this process is dependent on p63 which functions as a rheostat to control MEC fate choices, lineage fidelity, and cellular differentiation programs.

## Results

### ΔNp63 maintains the myoepithelial cell lineage in the adult mouse submandibular gland

**Fig. 1:**
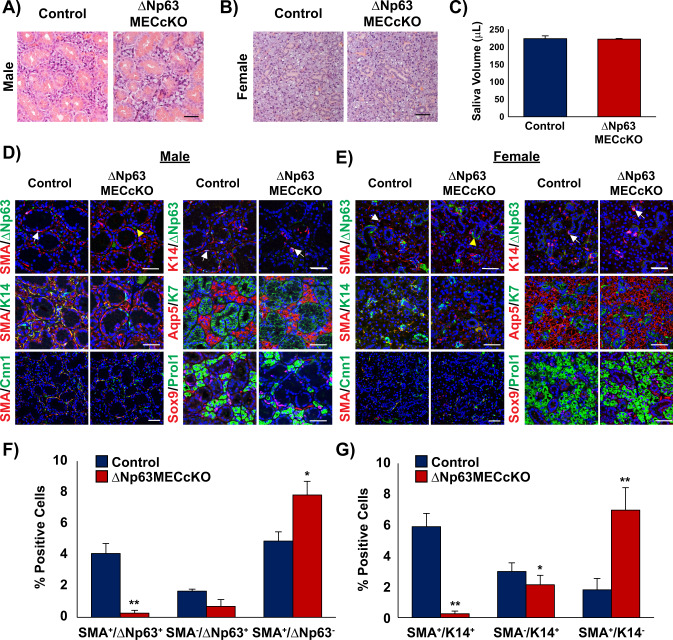
Histological and immunochemical analysis of submandibular salivary glands of mice with targeted deletion of ΔNp63 in the MECs. Hematoxylin and eosin (H&E) staining of **A** male and **B** female control and ΔNp63MECcKO submandibular glands 2 months post ΔNp63 deletion. **C** Saliva volume of the control and ΔNp63MECcKO mice 2 months post TAM administration. Immunochemical analysis of **D** male and **E** female control and ΔNp63MECcKO salivary glands show that the SMA^+^/ΔNp63^+^ and SMA^+^/K14^+^ double positive myoepithelial cell subpopulation decreases upon deletion of ΔNp63 in the MECs. Deletion of ΔNp63 in the myoepithelial cells does not appear to affect the other cell lineages as the acinar and ductal markers remain unchanged. White arrows indicate SMA^+^/ΔNp63^+^ MECs or ΔNp63^+^/K14^+^ basal cells. Yellow arrows depict background staining. Myoepithelial and basal cell marker: SMA, Cnn1, ΔNp63, K14; acinar cell marker: Aqp5, Prol1; ductal cell marker; K7, Sox9. Scale bar: 50 µm. (*n* = 4). **F** Quantification of SMA and ΔNp63 positive cells upon targeted deletion of ΔNp63. (*n* = 3). **G** Quantification of SMA and K14 positive cells upon targeted deletion of ΔNp63. Data are represented as mean ± standard deviation (SD) (*n* = 3). *p < 0.05, **p < 0.01. White arrows indicate p63^+^ cells, yellow arrows indicate non-specific p63 staining. Immunofluorescence staining colors: Yellow-green and red co-localization, Pink-red and blue (nuclei) co-localization, Turquoise-green and blue co-localization.

To examine the role of ΔNp63 in the myoepithelial cell population of the adult SMG, we crossed *ΔNp63*^*fl/fl*^ mice to transgenic animals that express CreERT2 under the control of *Acta2* regulatory elements (*Acta2*^*CreERT2*^) [26, 31]. Tamoxifen (TAM) was administered to adult male and female *ΔNp63*^*fl/fl*^ (control) and *Acta2*^*CreERT2*^*;ΔNp63*^*fl/fl*^ knockout (ΔNp63MECcKO) mice to induce targeted deletion of ΔNp63 selectively in the MECs. We harvested SMGs 2 months post TAM administration and performed histological analysis. Examination of hematoxylin and eosin (H&E) stained SMGs of the control and ΔNp63MECcKO mice did not show any noticeable alterations to the acinar or ductal structures in the male or female glands (Fig. 1A, B). In agreement with the normal appearance of the ΔNp63MECcKO SMGs, we did not observe any differences in saliva production in the knockout (KO) glands compared to the control (Fig. 1C). To determine if there were any phenotypic alterations resulting from the loss of ΔNp63 in the MECs, we next performed immunofluorescence studies of male and female control and ΔNp63MECcKO SMGs utilizing a panel of well-established epithelial cell markers. We began by confirming the loss of ΔNp63 protein expression in the MECs by co-staining the glands with ΔNp63 and SMA. As expected, we observed a significant decrease in the number of SMA^+^/ΔNp63^+^ double positive MECs in the KO SMGs compared to controls (Fig. 1D, E). Indeed, quantification of the various ΔNp63^+^ and SMA^+^ MEC populations corroborated our findings, further confirming loss of ΔNp63 expression in the MECs (Fig. 1F). Given the significant loss to the SMA^+^/ΔNp63^+^ MECs, we wondered whether this phenotype was a result of apoptosis due to ablation of ΔNp63 in this cell population. However, staining of control and ΔNp63MECcKO SMGs for the apoptotic marker cleaved caspase-3 (Casp3), revealed no significant differences at 1 month or 2 months post-TAM administration (Supplementary Fig. 1), suggesting possible alternate mechanisms, such as impaired self-renewal. Interestingly, while evaluation of the MEC-specific markers SMA and Calponin 1 (Cnn1) [32] revealed no noticeable differences in protein expression pattern between control and ΔNp63MECcKO SMGs (Fig. 1D, E), we did observe changes in K14 protein expression patterns in the MECs of the KO glands with ΔNp63^+^/K14^+^ basal cells still remaining (Fig. 1D, E, G, Supplementary Fig. 2A). In agreement with the histological findings, immunofluorescence staining for the ductal marker Keratin 7 (K7), showed no discernable alterations in the KO glands when compared to control counterparts (Fig. 1D, E). Finally, no changes were found in the acinar cells as revealed by Aquaporin 5 (Aqp5) staining which showed normal localization to the apical surface in both control and KO glands (Fig. 1D, E).

**Fig. 2:**
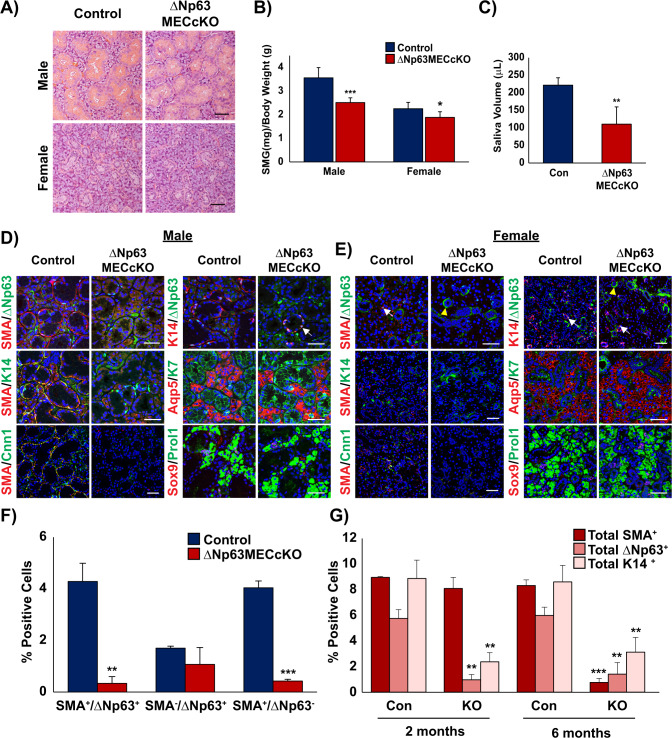
Histological and immunochemical analysis of submandibular salivary glands 6 months post ΔNp63 deletion in the MECs. **A** H&E staining of male and female control and ΔNp63MECcKO SMGs 6 months post ΔNp63 specific deletion in the MECs. **B** Submandibular gland weights of the male and female control and ΔNp63MECcKO mice 6 months post ΔNp63 ablation. **C** Saliva volume was measured from control and ΔNp63MECcKO mice 6 months following TAM administration. Immunofluorescence staining of **D** male and **E** female control and ΔNp63MECcKO SMGs demonstrates the dramatic loss of the MECs as shown by loss of SMA and Cnn1 expression. Basal, acinar, and ductal cell populations are still present in the ΔNp63MECcKO SMGs. White arrows show SMA^+^/ΔNp63^+^ MECs or ΔNp63^+^/K14^+^ basal cells. Yellow arrows depict background staining. Scale bar: 50 µm. **F** Quantification of SMA^+^ and ΔNp63^+^ cells 6 months post-TAM administration, shows a significant loss of the myoepithelial cell lineage. (*n* = 3). **G** Percentage of the total SMA, p63, K14 positive cells per total number of cells (total nuclei) in the control and ΔNp63MECcKO glands 2 months and 6 months post ΔNp63 MEC deletion. Data are represented as mean ± standard deviation (SD) (*n* = 3). *p < 0.05, **p < 0.01, ***p < 0.001. White arrows indicate p63^+^ cells, yellow arrows indicate non-specific p63 staining. Immunofluorescence staining colors: Yellow-green and red co-localization, Pink-red and blue (nuclei) co-localization, Turquoise-green and blue co-localization.

In light of the reported slow turnover rate of cells in the SMG [33, 34], we extended our analysis to 6 months post ΔNp63 deletion. While histological analysis failed to reveal any obvious alterations between KO and control glands, gross effects of the loss of ΔNp63 in the MECs were apparent (Fig. 2A, B). Interestingly, we found a reduction in the weights of both the male and female KO glands compared with the controls (Fig. 2B). This was accompanied with reduced saliva production in the KO glands, indicating a functional consequence due to loss of ΔNp63 (Fig. 2C). Next, to better appreciate the cellular alterations associated with the loss of ΔNp63 after 6 months, we co-stained male and female glands with ΔNp63 and SMA. Similar to the 2-month glands, we observed a significant reduction in the number of SMA^+^/ΔNp63^+^ double positive MECs in the KO SMGs compared to control with the majority of the ΔNp63^+^ cells localized to the basal cells surrounding the ducts (Fig. 2D–F). Similar findings were also observed in the sublingual and parotid glands suggesting that the specific consequences of loss of ΔNp63 were shared by all three major SGs (Supplementary Fig. 3). Strikingly, by 6 months, there was a dramatic loss to the MEC population in the KO glands as evident by co-staining of SMA and Cnn1 (Fig. 2D–G). In addition, we observed a decrease in the overall K14 protein expression levels in the MECs of the KO glands, with prominent expression mainly limited to the remaining ΔNp63^+^/K14^+^ basal cells (Fig. 2D, E, G, Supplementary Fig. 2B). While co-staining of the KO and control glands with K7 and Aqp5 did not show any significant alterations to the ductal or the acinar cells (Fig. 2D, E), a closer examination of the granular convoluted tubule (GCT) specific marker Mucin 13 (Muc13), revealed sex-specific differences as the female KO glands showed reduced protein expression levels compared to the control (Supplementary Fig. 4). To further validate our findings, we performed quantitative reverse-transcription polymerase chain reaction (qRT-PCR) for a select panel of genes that mark the various cell types in the control and ΔNp63MECcKO SMGs. Concordant with our immunostaining results, we confirmed reduced mRNA expression levels for a number of genes expressed in the MECs of both male and female KO mice including *Acta2*, *Myh11*, *Myl9*, and *Cnn1* (Supplementary Fig. 5). As expected, Δ*Np63* and its established targets, basal Keratins *Krt5* and *Krt14* [35], were also downregulated. While we did not observe significant alterations to marker genes expressed in the acinar cells, such as *Aqp5* and *Bhlha15*, the GCT marker *Muc13* was significantly downregulated in the female glands, in good agreement with our immunostaining results (Supplementary Fig. 5B). Taken together, these results confirm that ΔNp63 expression is crucial in maintaining multiple MEC sub-populations and highlights the importance of ΔNp63 in the MEC differentiation program.

### *Acta2* is a potential ΔNp63 target gene

**Fig. 3:**
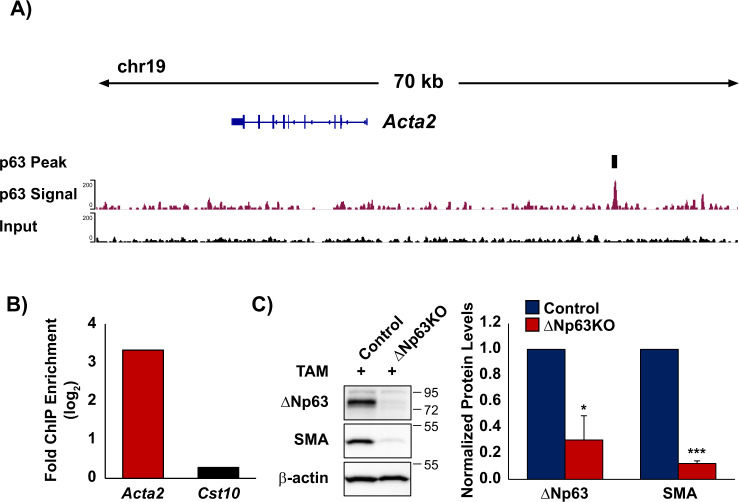
Regulation of *Acta2* expression by ΔNp63. **A** Visualization of the ΔNp63 binding site identified upstream of the *Acta2* genomic locus. Top line depicts the p63 binding site (black box). Bottom two lines display p63 ChIP signal enrichment, relative to input. **B** Independent ChIP-qPCR results using a ΔNp63 antibody in primary salivary gland epithelial cells confirm binding to the *Acta2* locus. *Cst10* serves as a negative control. Values represent mean fold enrichment over the random genomic locus, *Cst10*. **C** Representative western blot analysis of control and ΔNp63KO primary salivary gland epithelial cells were treated with activated tamoxifen (4-OHT) and analyzed by immunoblotting (left panel). The densitometric analysis of the western blot is shown on the right panel. ΔNp63 and SMA protein expression were normalized to β-actin. Data are represented as mean ± SD. (*n* = 3). *p < 0.05, ***p < 0.001.

We have previously shown that during SMG morphogenesis, SMA^+^ MECs are established from ΔNp63^+^ cells, similar to what has been reported in the lacrimal glands [11, 36]. However, the underlying molecular mechanisms directing the MEC lineage fate remain less understood. The dramatic loss to the MEC population upon ablation of ΔNp63 together with the downregulation of *Acta2* gene expression levels prompted us to examine if *Acta2* itself might be a transcriptional target of ΔNp63. Towards this end, we mined previous ΔNp63 ChIP-sequencing (ChIP-seq) datasets on salivary gland epithelial cells generated from our laboratory and identified a ΔNp63-binding region upstream of the *Acta2* genomic locus, suggesting that *Acta2* may be a transcriptional target of ΔNp63 (Fig. 3A). To confirm our ChIP-seq results, we next performed an independent ChIP followed by quantitative PCR (qPCR) quantification. Indeed, our ChIP-qPCR demonstrated selective enrichment of the putative p63 response element in the *Acta2* genomic locus compared to an intragenic region within the *Cst10* gene which served as a control (Fig. 3B). Armed with this information, we next performed knockdown (KD) experiments in primary SMG cells isolated from control (*ΔNp63*^*fl/fl*^) and ΔNp63KO mice, in which *ΔNp63*^*fl/fl*^ animals were crossed to a transgenic strain that ubiquitously expresses CreERT2 (*UBC*^*CreERT2*^*;ΔNp63*^*fl/fl*^). Primary SMG cells were treated with activated TAM to induce deletion of ΔNp63 expression, as previously reported [21]. Western blot analysis revealed reduced protein expression levels of ΔNp63 and SMA in the ΔNp63KO cells as compared to control cells (Fig. 3C). Taken together, these results suggest that ΔNp63 regulates the expression of *Acta2* in the SMG and that loss of ΔNp63 attenuates the levels of *Acta2* in vivo, likely through a transcriptional mechanism.

### ΔNp63 functions as a gatekeeper of the MEC differentiation program

**Fig. 4:**
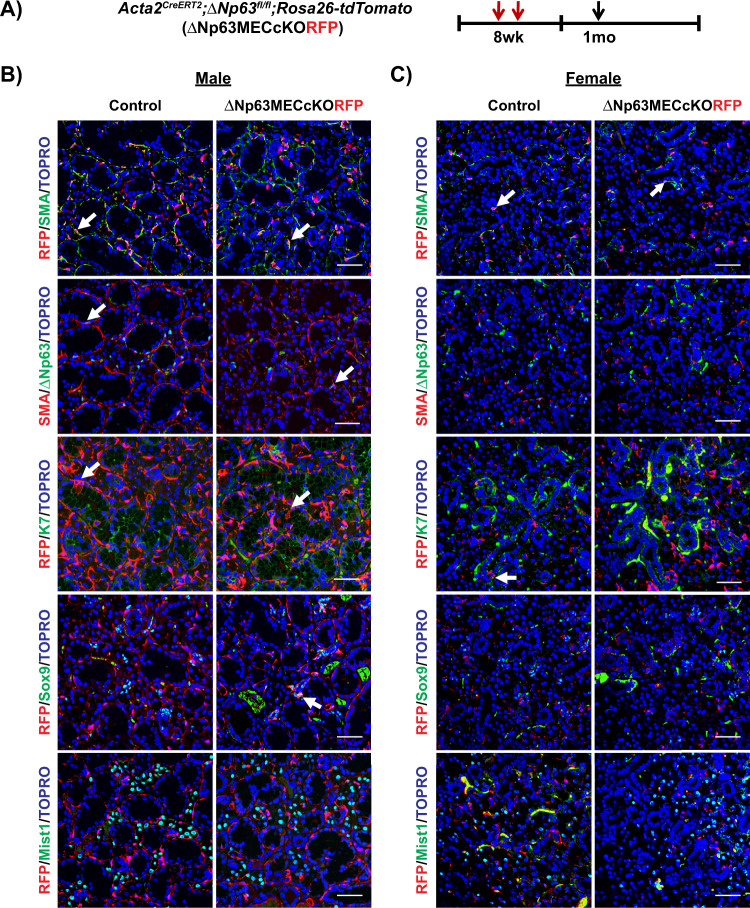
Lineage tracing of MECs 1 month post ΔNp63 deletion in this cell population under homeostatic conditions. **A** Experimental timeline used for lineage tracing of myoepithelial cells upon deletion of ΔNp63. Animals were administered TAM to simultaneously induce ΔNp63 specific deletion in the MECs and irreversibly label MECs with RFP expression. Animals were traced for 1 month and SMGs were analyzed. Immunostaining of the **B** male and **C** female control and ΔNp63MECcKORFP glands. Arrows indicate co-localization of RFP-positive cells with various epithelial cell markers. Myoepithelial: SMA, ΔNp63, Ductal: K7, Sox9, Acinar: Mist1. Scale bar: 50 µm.

**Fig. 5:**
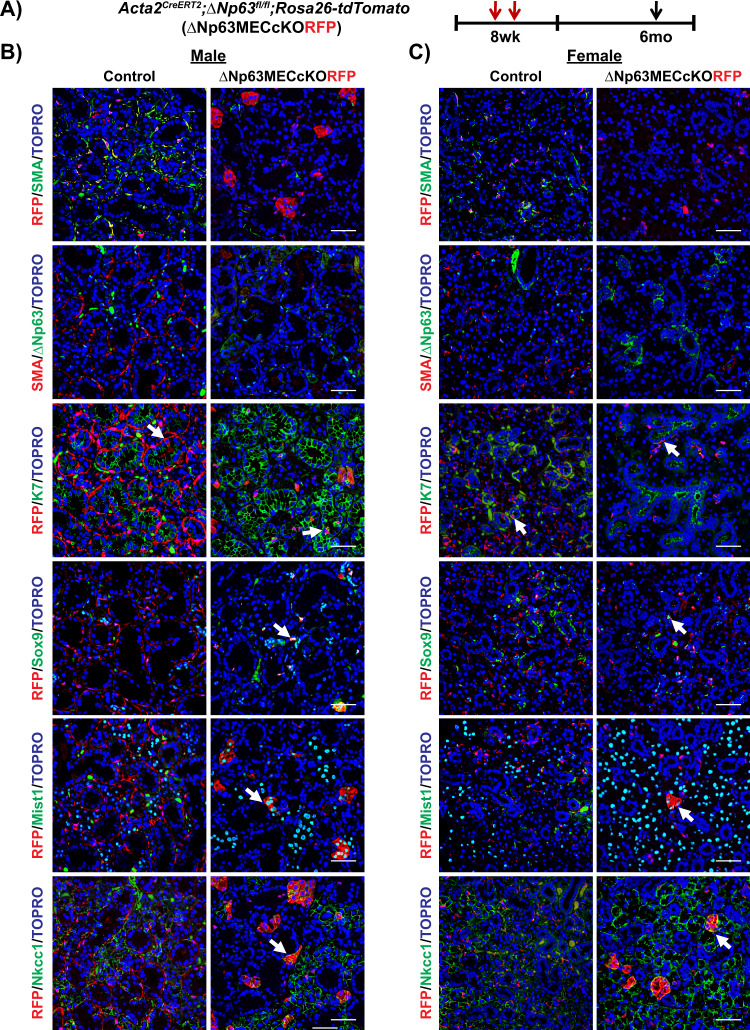
Lineage tracing of MECs 6 months post ΔNp63 deletion under homeostatic conditions. **A** Experimental timeline used for lineage tracing of myoepithelial cells upon deletion of ΔNp63. Animals were administered TAM to simultaneously induce ΔNp63 specific deletion in the MECs and irreversibly label MECs with RFP expression. Animals were traced for 6 months and SMGs were analyzed. Immunostaining of the **B** male and **C** female control and ΔNp63MECcKORFP glands. Arrows indicate co-localization of RFP-positive cells with various epithelial cell markers. Scale bar: 50 µm.

We have previously shown through genetic lineage tracing studies in the adult SMG, that under homeostatic conditions SMA^+^ MECs are bipotent and are able to maintain the ductal and MEC lineages [11]. Based on these findings, we wondered if ΔNp63 may play a role in directing MEC fate choices in adult SMG gland maintenance. To address this, we crossed the *Acta2*^*CreERT2*^*;Rosa26-tdTomato* mice to the Δ*Np63*^*fl/fl*^ mice to generate *Acta2*^*CreERT2*^*;*Δ*Np63*^*fl/fl*^*;Rosa26-tdTomato* knockout (ΔNp63MECcKORFP) animals. This allowed us to utilize Red Fluorescent Protein (RFP) expression as a surrogate marker to mark and trace MECs in which ΔNp63 expression has been specifically ablated [26]. To confirm efficiency and specificity of RFP labeling in the SMG, 8-week-old *Acta2*^*CreERT2*^*;Rosa26-tdTomato* mice were administered TAM, and SMGs were harvested after 1 day. Immunofluorescence analysis revealed robust and specific co-localization of RFP expression with SMA in the MECs of the SMG with an efficiency of approximately 73% (Supplementary Fig. 6). Based on these findings, 8-week-old ΔNp63MECcKORFP male and female mice were administered TAM and SMGs were harvested at two-time points; 1 and 6 months. Immunostaining of SMGs 1-month post-TAM administration revealed robust co-localization of RFP expression in the SMA^+^ MECs of the control glands and the ΔNp63MECcKORFP SMGs (Fig. 4). However, by 6 months, RFP^+^/SMA^+^ double positive cells were not detected in the KO glands compared to the control (Fig. 5). Notably, after 6 months we also observed a loss of SMA^+^/ΔNp63^+^ double-positive cells in the KO glands compared to control mice, confirming the specific deletion of ΔNp63 in the MECs (Fig. 5B, C). Similar to what we have previously reported, RFP^+^ cells co-localized to the K7^+^ ductal cells in the control glands reaffirming that MECs maintain the ductal cell population (Figs. 4B, C, 5B, C) [11]. Interestingly, the ability of SMA^+^ cells to maintain the K7^+^ ductal cell lineage was independent of ΔNp63 expression in the MECs as we observed RFP^+^ cells co-localized to the K7^+^ ductal cells in the KO glands at both time points examined (Figs. 4B, C, 5B, C). However, this was not the case for the intercalated ducts (ID) as we observed aberrant RFP expression in the Sox9^+^ IDs of the KO glands compared to control glands, suggesting that ΔNp63 is required for proper MECs differentiation (Figs. 4B, 5B). The quantification of the percentage of RFP^+^/Sox9^+^ double positive cells is shown in Supplementary Fig. 7. In contrast, while we did not detect any RFP expression in the Mist1^+^ acinar cells in control or KO glands 1-month post-TAM administration, after 6 months, we observed a significant number of RFP^+^/Mist^+^ double-positive cells in the KO glands, suggesting possible lineage infidelity (Figs. 4B, C, 5B, C). Quantification of the percentage of RFP^+^/Mist1^+^ double positive cells is shown in Supplementary Fig. 8. These findings were confirmed by co-staining of both male and female 6-month KO glands with an additional acinar-specific marker Na^+^/K^+^/2Cl^−^ co-transporter (Nkcc1) (Fig. 5B, C). Taken together, the altered cellular phenotypes and differentiation observed upon the loss of ΔNp63 in the MECs, suggests that this transcription factor may function as a critical checkpoint in directing proper MEC differentiation programs in the SMG during homeostasis.

### ΔNp63 is dispensable for MEC ability to respond during SMG regeneration

To ascertain the specific role of ΔNp63 in the MECs during regeneration, we utilized the duct ligation injury model which has been widely used to study SMG regeneration and injury repair mechanisms [37]. We began our analysis by examining the contribution of the SMA^+^ MECs in response to injury and regeneration by treating 8-week-old female *Acta2*^*CreERT2*^*;Rosa26-tdTomato* mice with TAM. A week after TAM treatment, animals were subjected to unilateral ligation of the main excretory duct with, or without, associate vasculature and nerves resulting in severe or mild injury, respectively [2, 13, 19]. The contralateral gland served as an uninjured control. After 14 days, the ligation was removed, and the glands were allowed to regenerate for 2 weeks post-de-ligation (Supplementary Fig. 9A). Staining of the glands with H&E showed dilation of the ductal lumens and a loss of the acinar cell population after 14 days of ligation (de-ligated) in both mild and severe injury (Supplementary Fig. 9B). Upon removal of the ligation followed by 14 days of regeneration (regenerated), acinar cells were replaced regardless of the extent of injury, however, there was a slower reappearance of acinar cells upon severe injury as previously described [2, 13, 19, 38] (Supplementary Fig. 9B). To evaluate the contribution of SMA^+^ cells to repair, regenerated *Acta2*^*CreERT2*^*;Rosa26-tdTomato* glands were co-stained with RFP and K7 or Mist1 to examine the state of the ducts and acinar cells, respectively. We found that RFP^+^ cells were present in the ducts (K7) and acinar cells (Mist1) of the glands that were severely injured, while the mildly injured glands only showed RFP expression in the ductal cells, similar to what we had observed under homeostatic conditions (Fig. 4), and in agreement with what has been reported in the literature (Supplementary Fig. 9C) [19].

**Fig. 6:**
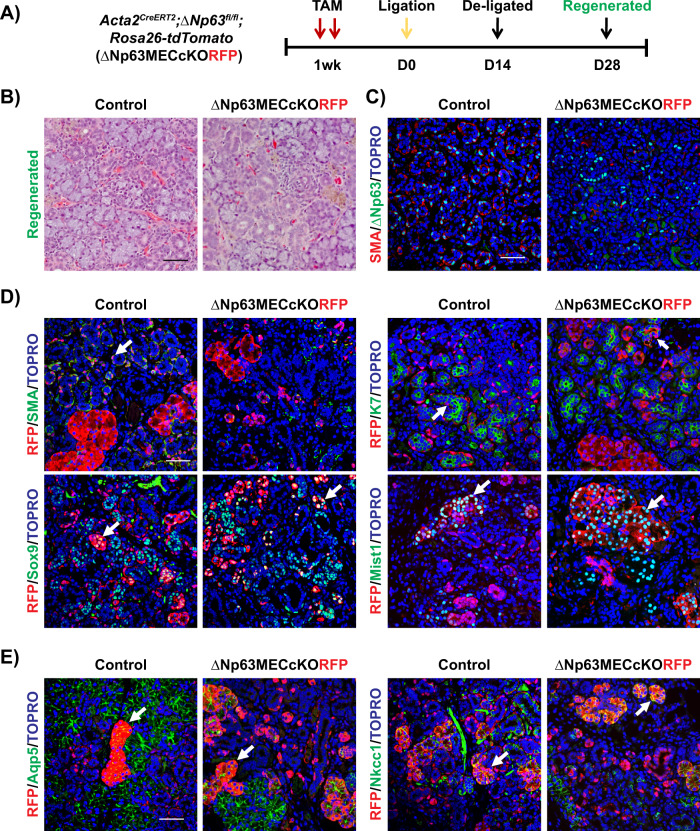
Contribution of ΔNp63 ablated MECs to SMG regeneration following severe injury. **A** Experimental timeline used to collect the regenerated SMGs of the control and ΔNp63MECcKORFP mice. **B** Histological analysis of the regenerated female control and ΔNp63MECcKORFP glands. **C** Immunostaining images of regenerated KO glands stained with the MEC markers SMA and ΔNp63. **D** Immunochemical analysis shows that RFP co-localizes with K7, Sox9 (ductal marker), and Mist1 (acinar marker) in both the control and ΔNp63MECcKORFP regenerated SMGs. RFP^+^ cells represent the progeny of the SMA^+^ myoepithelial cells. **E** RFP^+^ cells co-localize with Aqp5 and Nkcc1 (acinar markers) in the control and ΔNp63MECcKORFP-regenerated SMGs. White arrows indicate co-localization of RFP and specific cell population markers. Scale bar: 50 µm.

Given that the progeny of SMA^+^ RFP MECs were detected in the ductal and acinar cells following severe injury, we next evaluated the requirement of ΔNp63 in the MECs during regeneration following severe injury. Towards this end, 8-week-old female control and ΔNp63MECcKORFP mice were administered TAM to delete ΔNp63 and after 1 week, animals were subjected to ductal ligation (Fig. 6A). Histological analysis confirmed regeneration in both the control and KO glands as observed by the re-appearance of acinar cells (Fig. 6B). As expected, there was a dramatic loss of the SMA^+^/ΔNp63^+^ double positive MECs (Fig. 6C). Interestingly, lineage traced regenerated glands revealed RFP expression in the ducts and acinar cells of the ΔNp63MECcKORFP and control mice as demonstrated by co-localization of RFP with the ductal (K7) and acinar (Mist1, Aqp5, and Nkcc1) specific markers (Fig. 6D, E). Collectively, these results suggested that, unlike SMG homeostasis, the contribution of MECs to ductal and acinar cell regeneration following severe injury is independent of ΔNp63.

## Discussion

**Fig. 7:**
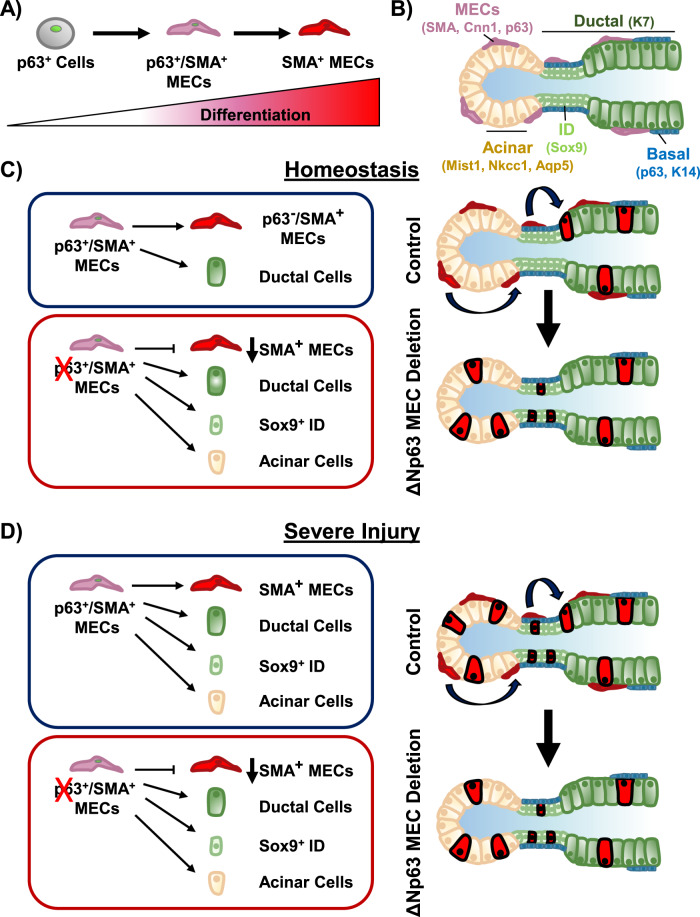
Schematic outlining the myoepithelial cell differentiation program and the contribution of MECs to the salivary gland during homeostatic and regenerative states. **A** p63 expressing cells give rise to SMA^+^/p63^+^ double positive MECs, which further differentiate into SMA^+^/p63^−^ MECs. **B** Structure of the salivary gland and cell markers that represent the various cell types that comprise the salivary gland. **C** During homeostasis the myoepithelial cells are bipotent and maintain their own lineage (MECs) and the ductal cell lineage. Deletion of ΔNp63 in the MECs leads to altered cellular differentiation and plasticity and a failure in MEC renewal. **D** After severe injury, SMA positive cells undergo a bipotency to multipotency switch and contribute to the ductal and acinar cell populations. This process is ΔNp63 independent.

ΔNp63 is enriched in the stem/progenitor cell populations of epithelial tissues including the SMG where it has been shown to play critical roles in directing cell fate choices and differentiation programs. Indeed, systemic deletion of ΔNp63 in the adult mouse SMG have revealed the critical role for this transcriptional regulator in directing acinar and ductal cell differentiation programs as well as maintaining the stem/progenitor cell populations [21]. While these studies have been important in understanding the widespread effects of loss of p63 in the SMG, investigations focused on teasing out the more nuanced cell-type specific functions of this factor, particularly as it pertains to the MECs, have been lacking. Here we have performed multiple genetic mouse model-based studies to showcase a novel role for ΔNp63 in maintaining the MEC population in adult SMGs, in part through regulation of *Acta2* gene expression. We further demonstrate that during SMG homeostasis, SMA^+^ MECs function as bipotent stem/progenitor cells that maintain the MEC and ductal cell lineages. However, ΔNp63 specific deletion in the MECs results in altered MEC differentiation and triggers heightened cellular plasticity allowing for their differentiation to intercalated ducts and acinar cells, suggesting that ΔNp63 functions as an important gatekeeper of the MEC differentiation program (Fig. 7).

Prior studies involving systemic deletion of ΔNp63 targeting both p63 expressing basal and MECs revealed a complete loss of the stem/progenitor cell populations along with striking changes to the ductal and acinar cell differentiation programs of the SMG [21]. Conversely, MEC selective ablation of ΔNp63 resulted in the loss of the MECs with no discernable changes to the other epithelial cell types. Based on these findings, it is tempting to speculate that p63^+^ basal cells and p63^+^ MECs may have non-redundant roles in SMG biology. This notion is supported by lineage tracing experiments of p63^+^ cells in the SMG which have demonstrated the multipotency of these cells as they were shown to maintain the basal, ductal, myoepithelial, and to a lesser extent, acinar cell populations [11]. Conversely, results of lineage tracing studies of SMA^+^ MECs, which contain a population of p63^+^/SMA^+^ double positive cells, have revealed these cells to be bipotent by maintaining the MEC and ductal cell lineages only [11]. These findings are suggestive of a continuum of p63 stem/progenitor cell states in which multipotent p63^+^ cells may differentiate to bipotent p63^+^/SMA^+^ MECs (Fig. 7A). Additional experiments focused on teasing out the contribution of the various p63^+^ cell populations in the SMG will be required.

While our SMG lineage tracing studies reported here have demonstrated the bipotency of SMA^+^ MECs in maintaining their own cell population as well as contributing to the ducts during homeostasis, knockout-based results suggest this process to be p63-dependent. Indeed, tracing of the ΔNp63-null MECs during homeostasis revealed these cells to undergo a bipotency to multipotency switch in which the ΔNp63-null MECs expanded their differentiation capabilities to include both IDs and acinar cells (Fig. 7C). These results highlight the important role of p63 in restricting the cellular differentiation and innate plasticity of the epithelial cells of the SMG and its ability to function as a molecular switch of cell fate, similar to what has been reported recently in the lung [39]. Interestingly, we found that in contrast to mild injury, upon severe injury, the MEC switch from bipotency to multipotency occurred regardless of p63 expression (Fig. 7D). This MEC switch to bipotency is similar to recent findings in the SMG and the airway surface epithelium [19, 40, 41]. Follow-up studies to elucidate the p63-driven mechanisms that direct epithelial cell differentiation and/or lineage choices in the MECs will be needed.

An interesting phenotype we observed was the significant loss of saliva volume in the ΔNp63MECcKO mice 6-months post-TAM administration. This is likely due to the loss of the MEC population in the KO animals and reminiscent of the mammary gland secretory defects observed in *Acta2*-null female mice [42–44]. However, we cannot rule out the possibility that the reduced saliva volume may also result in part, due to a functional deficiency of the acinar cells secondary to the loss of the MECs. Indeed, studies of the mammary gland have demonstrated that a p63-dependent paracrine cell signaling axis exists between the MECs and the luminal cells that orchestrates the entire lactation program [45]. Whether similar crosstalk mechanisms are in the play between p63^+^ MECs and the acinar cell populations of the SMG will be an interesting avenue for future studies. Additionally, the eventual loss of the MECs in the ΔNp63MECcKO animals was another intriguing and unexpected finding. While studies in the skin and mammary gland have shown the role of ΔNp63 in directing basal cell identity, this is the first report demonstrating an indispensable role for ΔNp63 in not only establishing MEC fate identity, but also in maintaining/renewing this distinct cell population. We posit that ΔNp63 might direct and/or promote MEC fate by regulating *Acta2* gene expression although sustained expression of *Acta2* in the adult SMG does not depend upon ΔNp63. This might involve other transcription factors and signaling pathways, such as YAP and TAZ that operate in MECs, as recently reported [46].

Overall, our study provides insight into the p63-driven MEC differentiation programs important for proper SMG homeostasis and regeneration. Our in vivo lineage tracing experiments show that under homeostatic conditions, SMG MECs function as a bipotent stem/progenitor cell population that maintains the myoepithelial and ductal cell lineages. We further demonstrate that this process is dependent on the transcription factor ΔNp63 which works as a molecular switch to control MEC fate choices and drive cellular differentiation. While ΔNp63 is dispensable for the response of MECs to SMG regeneration after severe injury, it is critical for maintaining/renewing this important cell population under homeostatic conditions. Additional studies aimed at uncovering signals and regulatory players that control cell fate decision and differentiation programs in the context of SMG homeostasis and regeneration after injury will be critical and are likely to yield important information aimed at developing new strategies to treat hyposalivation.

## Materials and Methods

### Animal experiments

All animal experiments and procedures were performed in accordance with the State University of New York at Buffalo (University at Buffalo) Institutional Animal Care and Use Committee (IACUC) regulations. All procedures were approved by University at Buffalo IACUC. C57BL/6J (Stock No. 000664), *Rosa26-tdTomato* (B6.Cg-*Gt(ROSA)26Sor*^*tm14(CAG-tdTomato)Hze*^/J; Stock No. 007914), and *UBC*^*CreERT2*^ (B6.Cg-Ndor1^Tg(UBC-cre/ERT2)1Ejb^/1J; Stock No. 007001) mice were purchased from The Jackson Laboratory (Bar Harbor, Maine). *Acta2*^*CreERT2*^ mice were provided from Pierre Chambon and ΔNp63-floxed (*ΔNp63*^*fl/fl*^) mice were provided by Elsa Flores and have been described previously [26, 31]. All mice were maintained on a C57BL/6J background. To induce Cre-loxP recombination for knockout studies and lineage tracing analysis, the inactive form of tamoxifen (TAM; Sigma-Aldrich, T-5648) was dissolved in corn oil, and 2 mg of TAM was intraperitoneally (IP) injected to 8-week adult mice twice as previously described [11]. Animals were euthanized by CO_2_ inhalation and the salivary glands were further dissected at specific time points of interest. Sample sizes were determined according to the standard protocols in the field. No criteria was set for excluding mice and no blinding to group allocation was performed.

### Duct ligation

One week before the duct ligation surgery, female Δ*Np63*^*fl/fl*^, *Acta2*^*CreERT2*^*;*Δ*Np63*^*fl/fl*^(ΔNp63MECcKO), *Acta2*^*CreERT2*^*;Rosa26-tdTomato*, and *Acta2*^*CreERT2*^*;*Δ*Np63*^*fl/fl*^*;Rosa26-tdTomato* (ΔNp63MECcKORFP) mice were intraperitoneally (IP) injected at 8 weeks with TAM. The mice were weighed and anesthetized by IP injection with ketamine (80 mg/kg) and xylazine (10 mg/kg) and the main excretory duct was ligated with or without the associated blood vessels with a titanium hemostatic clip (Vitalitec Int., R9180) to induce ligature-induced injury. Two weeks post-ligation, the titanium metal clip was removed or the salivary gland was dissected and fixed in 10% Neutral Buffered Formalin (NBF) for further analysis (“De-ligated” stage). Following an additional two weeks after de-ligation, the submandibular glands were dissected and fixed in 10% Neutral Buffered Formalin (NBF) for further analysis as the “Regenerated” stage. (*n* = 3).

### Salivary gland weight

The submandibular gland weight (mg) was measured and normalized by the mouse body weight (g). (*n* = 7).

### Saliva collection

Saliva was harvested from Δ*Np63*^*fl/fl*^ (control) and *Acta2*^*CreERT2*^*;*Δ*Np63*^*fl/fl*^(ΔNp63MECcKO) mice for 10 min following intraperitoneal injection with pilocarpine HCl (0.3 mg/100 µL, Sigma-Aldrich). Saliva volume was measure by a pipette. (*n* = 5).

### Immunostaining and imaging

Salivary glands were fixed in 10% NBF and processed for paraffin embedding. Paraffin-embedded samples were sectioned at 5 μm using charged slides (Fisherbrand Colorfrost Plus Microscope slides). Salivary gland sections were deparaffinized and rehydrated through a graded series of alcohol and Phosphate Buffered Saline (PBS) and further used for Hematoxylin and Eosin (H&E) staining or immunofluorescence staining. H&E staining was performed by staining the deparaffinized slides with hematoxylin (Sigma-Aldrich, GHS316) and eosin (Richard-Allen Scientific). H&E images were taken using an Axio microscope (200X). For immunofluorescence analysis, antigen retrieval was performed with sodium citrate buffer (10 mM sodium citrate, 0.05% Tween-20, pH6) in a pressure cooker for 10 min. Slides were rinsed briefly in PBS and blocked using the Mouse on Mouse (M.O.M.) kit (Vector Laboratories). Primary antibodies used at the indicated dilutions include alpha-smooth muscle actin (SMA) (1:200, Sigma-Aldrich, 1A4), p63 (1:50, Cell Signaling Technology, D2K8X), K14 [47] (1:100), Aqp5 (1:100, Alomone Labs), K7 (1:50, Abcam), Sox9 (1:50, Cell Signaling Technology), Prol1 (1:100, Everest Biotech), Cnn1 (1:100, Sigma-Aldrich), Muc13 (1:100, Santa Cruz Biotechnology), Mist1 (1:100, Abcam), Nkcc1 (1:100, Cell Signaling Technology), Nkcc1 (1:100, Santa Cruz Biotechnology), RFP (1:100, Rockland), DsRed (1:50, Clontech), Cleaved Caspase 3 (Casp3; 1:100, Cell Signaling Technology). Sections were stained with TOPRO (Invitrogen) and mounted using VECTASHIELD Antifade Mounting Medium with DAPI (Vector Laboratories) and imaged using a ZEISS LSM 510 Meta Confocal microscope with ZEISS ZEN Black imaging software or an Andor Dragonfly Spinning Disk Confocal Microscope with Fiji [48]. Microscopy data in this study was acquired at the Optical Imaging and Analysis Facility, School of Dental Medicine, State University of New York at Buffalo. *n* = 6 mice per sex.

### Quantifications

All analyses were performed by using confocal images and quantified using Image J (NIH; Bethesda, Maryland). Quantified values were reported as mean ± standard deviation (S.D.) of three or more independent mice for each group.

### Quantification of SMA, ΔNp63, K14 positive cells

The submandibular glands were stained with SMA, ΔNp63, and K14 antibodies to quantify the various cell subpopulations. The SMA^+^/K14^+^ positive cells were quantified by counting the SMA^+^ single positive, K14^+^ single positive, and SMA^+^/K14^+^ double positive cells. These numbers were calculated into percentage based on the total nuclei. The SMA^+^/ΔNp63^+^ positive cells were quantified in a similar fashion. The total number of SMA^+^, ΔNp63^+^, and K14^+^ positive cells were calculated by quantifying the total positive cells, divided by the total nuclei, respectively. A minimum of five fields of view (40X magnification/objective) were used for each quantification analysis. (*n* = 3).

### Quantification of RFP-positive cells

The RFP^+^ cells were quantified as previously described [11]. Briefly, the percentage of the RFP^+^ cells that co-express a cell lineage marker (for example SMA, Sox9, Mist1) were calculated by quantifying the RFP^+^ and lineage marker double positive cells, divided by the total number of RFP^+^ cells (*n* = 3). Quantification analyses were performed using three to five fields of view (40X magnification/objective confocal images) using ImageJ (NIH; Bethesda, Maryland).

### Quantification of cell apoptosis

Cell apoptosis was calculated by quantifying the Casp3^+^ single positive or K7^+^Casp3^+^ double positive cells which were divided by the total number of nuclei or total ductal cell number (K7^+^), respectively (*n* = 3 for each time point). Quantification analyses were performed using three to five fields of view (40X magnification/objective confocal images) using ImageJ (NIH; Bethesda, Maryland).

### RNA isolation and quantitative RT-PCR

Total RNA was extracted from Δ*Np63*^*fl/fl*^ (control) and *Acta2*^*CreERT2*^*;*Δ*Np63*^*fl/fl*^ knockout (ΔNp63MECcKO) mouse submandibular glands 6 months following TAM administration were homogenized in Trizol reagent (Invitrogen) using BioMashers (TaKaRa). The RNA was phase separated by chloroform and further isolated using the Direct-zol RNA Miniprep kit (Zymo Research). cDNA was synthesized by reverse transcribing isolated RNA using the iScript cDNA Synthesis kit (Bio-Rad). qRT-PCR (quantitative Real-Time Reverse Transcription- Polymerase Chain Reaction) was performed as previously described [21]. Briefly, qRT-PCR was performed on a CFX96 Touch Real-Time PCR machine using iQ SYBR Green Supermix (Bio-Rad) in triplicates in at least three independent biological replicates. Relative expression values of each target gene were normalized to hypoxanthine guanine phosphoribosyltransferase (*Hprt*) expression. Primer sequences are shown in Supplementary Table 1. *n* = 3 for each sex.

### Chromatin immunoprecipitation-sequencing analysis and qPCR validation

The previously reported ChIP-sequencing (ChIP-seq) dataset (Gene Expression Omnibus (GEO) database under the accession number GEO: GSE145264) was mapped to the Mus musculus genome (mm9 build) and ChIP-seq signals were visualized using Integrative Genomics Viewer (IGV) [21, 49]. p63 ChIPed DNA was used for real-time qPCR (ChIP-qPCR) to validate binding to the *Acta2* genomic locus, using *Cst10* (which does not have a p63 binding site) as a negative control. The *Acta2* ChIP-qPCR primer sequences are shown in Supplementary Table 1.

### p63 in vitro knockdown assay and western blot analysis

Primary salivary gland epithelial cells were generated from Δ*Np63*^*fl/fl*^ (control) and *UBC*^*CreERT2*^*;*Δ*Np63*^*fl/fl*^ (ΔNp63KO) mouse submandibular glands and seeded on plastic 6 well plates (10^6^ cells/well) in CnT-Prime medium (CELLnTEC) and cultured at 37 °C in 5% CO_2_. The cells were treated with activated tamoxifen ((Z)-4-Hydroxytamoxifen, 4-OHT, Sigma-Aldrich) 11 days after plating in order to knock down ΔNp63. Three days after tamoxifen treatment, the cells were washed with PBS, lysed in RIPA buffer containing a protease inhibitor cocktail (G-Biosciences), and subjected to western blot analysis. Protein concentration was determined by using the Bio-Rad Bradford protein assay. The protein samples were separated by SDS-PAGE and transferred to a PVDF membrane and blocked with 5% non-fat dry milk in Tris Buffered Saline with Tween-20 (TBST). For primary antibodies, p63 (1:10,000, Cell Signaling Technology), SMA (1:10,000, Sigma-Aldrich), and β-actin (1:10,000, Cell Signaling Technology) were used. KPL LumiGLO Reserve Chemiluminescent Substrate kit (Sera care) was applied to the membrane and the ChemiDoc MP Imaging System (Bio-Rad) was use for detection. Densitometry (measurement of band intensity) was performed by using Image J (NIH) and p63 and SMA expression was normalized by β-actin. (*n* = 3).

### Statistical analysis

Quantified results were reported as mean ± standard deviation (SD) of three or more independent experiments or mice for each group. Two-tailed student’s *t*-test was used for comparison of two groups and the threshold for significance was set at *p* < 0.05. No randomization methods were used.

## Supplementary information

Supplementary Figures

aj-checklist
