# Supplementary material for: ΔNp63 maintains the fidelity of the myoepithelial cell lineage and directs cell differentiation programs in the murine salivary gland
Source: Cell Death Differ. 2022 Dec 16;30(2):515–26. doi: 10.1038/s41418-022-01101-0 (PMC9950458; doi:10.1038/s41418-022-01101-0)
Supplement: Supplementary file 1 — Supplementary Figures [file 41418_2022_1101_MOESM1_ESM.docx]

**ΔNp63 Maintains the Fidelity of the Myoepithelial Cell Lineage and Directs Cell Differentiation Programs in the Murine Salivary Gland**

**Eun-Ah Christine Song, Monika Che, Jason Osinski, Kirsten Smalley, Erich Horeth, Satrajit Sinha and Rose-Anne Romano**

**Supplementary Fig. 1. Evaluation of apoptosis in the submandibular glands upon deletion of ΔNp63 in the MECs.** Expression and quantification analysis of cleaved caspase-3 (Casp3) reveals no changes in cell apoptosis in the KO compared to control SMGs 1 month **A)** and **B)** 2 months post TAM administration. Arrows indicate Casp3^+^ cells. n=3. Scale bar: 50µm.

**Supplementary Fig. 2. Immunostaining analysis of the myoepithelial cell subtypes in the male submandibular glands upon targeted deletion of ΔNp63 in the MECs. A)** Top panel shows the experimental timeline used to generate *Acta2^CreERT2^;ΔNp63^fl/fl^* knockout (ΔNp63MECcKO) mice 2 months post-tamoxifen (TAM) administration. Lower panel shows expression of ΔNp63 in the SMG MECs of control and KO mice. ΔNp63 expression is lost in the MECs of the KO mice. **B)** Top panel shows the experimental timeline used to generate ΔNp63MECcKO mice 6 months post TAM administration. Lower panel shows expression of ΔNp63 in the SMG MECs in control and KO mice. MECs are lost in the KO mice. Scale bar: 50µm.

**Supplementary Fig. 3. Immunostaining analysis of the myoepithelial cells in the sublingual and parotid salivary gland 6 months post targeted deletion of ΔNp63 in the myoepithelial cells.** Top panel shows the experimental timeline used to generate ΔNp63MECcKO mice 6 months post TAM administration. Lower panel show expression of ΔNp63 in the sublingual gland and the parotid gland MECs of control and KO mice. MECs are lost in the KO mice. Scale bar: 50µm.

**Supplementary Fig. 4. Muc13 expression in the male and female glands 6 months post targeted deletion of ΔNp63 in the myoepithelial cells. A)** Experimental timeline used to generate ΔNp63MECcKO mice 6 months post TAM administration. Immunostaining analysis showing expression of the GCT marker Muc13, in the **B)** male and **C)** female ΔNp63MECcKO submandibular glands. Scale bar: 50µm.

**Supplementary Fig. 5. Transcriptomic analysis of ΔNp63MECcKO submandibular glands.** Quantitative RT-PCR analysis of the **A)** male and **B)** female ΔNp63MECcKO submandibular glands 6 months post TAM administration. Myoepithelial and basal genes: *ΔNp63*, *Acta2, Myh11, Myl9, Cnn1, Krt14, Krt5*, Acinar genes: *Aqp5, Bhlha15, Slc12a2*, Ductal genes: *Krt18, Krt19, Sox9, Muc13.* Data are represented as mean ± SD. (n=3). *p<0.05, **p<0.01, ***p<0.001.

**Supplementary Fig. 6. RFP expression in *Acta2^CreERT2^;Rosa26-tdTomato* mice 1 day post TAM administration.** Immunofluorescence imaging shows co-localization of RFP expression with SMA in the MECs (left panel). Right panel shows the quantification of RFP^+^/SMA^+^ double positive cells. (n=3). Scale bar: 50µm.

**Supplementary Fig. 7. Quantification of the myoepithelial derived Sox9 expressing cells.** Quantification of the percentage of the RFP^+^/Sox9^+^ cells in the control and ΔNp63MECcKORFP male submandibular glands 6 months post ΔNp63 deletion. Data are represented as mean ± SD. (n=3). **p<0.01

**Supplementary Fig 8. Quantification of the Mist1 expressing cells derived from the myoepithelial cells.** Quantification of RFP^+^ cells that express Mist1 6 months after deletion of ΔNp63 in the MECs. Data are represented as mean ± SD. (n=3).

**Supplementary Fig. 9. Lineage tracing myoepithelial cells after mild and severe injury. A)** Timeline outlining the ductal ligation experiments of the *Acta2^CreERT2^;Rosa26-tdTomato* mice. **B)** Histological analysis of the SMGs after mild injury (left panel) and severe injury (right panel) at the de-ligated and regenerated states. **C)** Contribution of RFP^+^ MECs in regenerated glands after mild injury (left panel) and severe injury (right panel). Arrows indicate co-localization of RFP positive cells with the ductal cell marker K7 or the acinar cell marker Mist1. Scale bar: 50µm.

**Supplementary Table 1.**

**Original Western Blot- Fig. 3C.**
